# Online dosimetric evaluation of larynx SBRT: A pilot study to assess the necessity of adaptive replanning

**DOI:** 10.1002/acm2.12019

**Published:** 2016-12-22

**Authors:** Weihua Mao, Timothy Rozario, Weiguo Lu, Xuejun Gu, Yulong Yan, Xun Jia, Baran Sumer, David L. Schwartz

**Affiliations:** ^1^ Department of Radiation Oncology University of Texas Southwestern School of Medicine Dallas TX USA; ^2^ Department of Radiation Oncology Henry Ford Hospital Detroit MI USA; ^3^ Department of Otolaryngology University of Texas Southwestern School of Medicine Dallas TX USA

**Keywords:** adaptive radiation therapy, delivery QA, larynx cancer, replanning, SBRT

## Abstract

**Purpose:**

We have initiated a multi‐institutional phase I trial of 5‐fraction stereotactic body radiotherapy (SBRT) for Stage III–IVa laryngeal cancer. We conducted this pilot dosimetric study to confirm potential utility of online adaptive replanning to preserve treatment quality.

**Methods:**

We evaluated ten cases: five patients enrolled onto the current trial and five patients enrolled onto a separate phase I SBRT trial for early‐stage glottic larynx cancer. Baseline SBRT treatment plans were generated per protocol. Daily cone‐beam CT (CBCT) or diagnostic CT images were acquired prior to each treatment fraction. Simulation CT images and target volumes were deformably registered to daily volumetric images, the original SBRT plan was copied to the deformed images and contours, delivered dose distributions were re‐calculated on the deformed CT images. All of these were performed on a commercial treatment planning system. In‐house software was developed to propagate the delivered dose distribution back to reference CT images using the deformation information exported from the treatment planning system. Dosimetric differences were evaluated via dose‐volume histograms.

**Results:**

We could evaluate dose within 10 minutes in all cases. Prescribed coverage to gross tumor volume (GTV) and clinical target volume (CTV) was uniformly preserved; however, intended prescription dose coverage of planning treatment volume (PTV) was lost in 53% of daily treatments (mean: 93.9%, range: 83.9–97.9%). Maximum bystander point dose limits to arytenoids, parotids, and spinal cord remained respected in all cases, although variances in carotid artery doses were observed in a minority of cases.

**Conclusions:**

Although GTV and CTV SBRT dose coverage is preserved with in‐room three‐dimensional image guidance, PTV coverage can vary significantly from intended plans and dose to critical structures may exceed tolerances. Online adaptive treatment re‐planning is potentially necessary and clinically applicable to fully preserve treatment quality. Confirmatory trial accrual and analysis remains ongoing.

## 1 Introduction

Squamous cell carcinoma of the larynx is common in North America.^1^ Organ preservation with chemoradiotherapy represents standard‐of‐care for locally advanced disease.^2^, ^3^ Conventional techniques deliver 70 Gy over 7 weeks with incidental coverage of uninvolved larynx and healthy bystander tissues. Long‐term outcomes from RTOG 91‐11 demonstrated comparable larynx preservation and overall survival with sequential or concurrent chemoradiotherapy.^4^ A discouraging finding from this trial was that improved organ preservation with concurrent chemoradiotherapy came at the cost of late deaths, potentially due to undetected toxicity. Since this trial, no significant advances have been made in radiation‐based organ preservation strategies for advanced larynx cancer.

Accelerated hypofractionated irradiation of early‐stage larynx cancer originated in Europe, with early results mirroring those achieved with conventional therapy.^5^, ^6^ Despite conventional radiation techniques, there was no difference in cure rates when reducing radiation therapy from a 5‐week course down to 3 weeks.^7^ Later, a British Institute of Radiology study showed equivalent survival rates and no significant differences in toxicity with either a 3‐week or 6‐week radiation course.^6^ More recently, the Royal Marsden Hospital treated 200 patients with T1 glottic cancer to a dose of 50–52.5 Gy in 16 daily fractions,^5^ matching outcomes from historical studies. Beyond patient convenience and cost‐saving advantages, hypofractionated radiation therapy may improve local control rates.^8^ A phase III clinical trial showed improved local control with 56.25 Gy in 25 fractions compared to 60–66 Gy in 30–33 fractions, with equivalent toxicity.^9^

We have initiated a multi‐institutional phase I trial of 5‐fraction stereotactic body radiotherapy (SBRT) for Stage III–IVa laryngeal cancer. SBRT divides intended radiation dose into five or fewer fractions with steep dose gradients and tight treatment accuracy constraints. Thus, SBRT employs dramatically higher daily doses than conventional therapy, and holds promise for improving outcomes for high‐risk disease. For example, local control of early‐stage lung cancer with conventional radiation treatment is less than 50%, while newer series employing SBRT demonstrate improved local control approximating 90%.^10^, ^11^, ^12^

Current radiation delivery techniques are based on a planning CT scan acquired before therapy begins, without planned changes during treatment. The geometry of tumor and normal anatomy can shift significantly secondary to movement and tissue responses. Serial CT studies taken during standard treatment demonstrate that tumors can shrink by > 90% during a 7‐week course of treatment, and that parotid glands can involute and shift by up to a centimeter.^13^ Adaptive replanning techniques have been leveraged to correct for these changes and are evolving toward becoming a routine component of standard‐of‐care.^14^, ^15^ To our knowledge, there are no published reports describing dosimetric variances which take place during accelerated hypofractionated treatment of head and neck cancer. In this report, we describe *post hoc* calculation of dose variances and pilot validation of an online adaptive planning platform to preserve treatment quality in a series of ten patients treated on prospective institutional clinical trials formally investigating SBRT for definitive treatment of laryngeal cancer.

## 2 Methods

For this pilot analysis we included five patient cases (Pt# 6–10) enrolled onto an advanced stage SBRT dose searching phase I trial (NCT02464137) and five patients (Pt# 1–5) enrolled onto a separate phase I SBRT trial for early‐stage disease (NCT01984502). All ten patients were treated at the same institution and their data were analyzed identically. Patient characteristics are described in Table 1. Gross Tumor Volume (GTV) was defined as all known gross disease determined by examination, CT, MRI, and FDG‐PET images. All equivocal radiographic abnormalities, such as clinically suspicious lymph nodes, were included within GTV. The Clinical Target Volumes (CTV) was defined as the GTV plus areas at risk for adjacent spreads of microscopic disease. The circumferential margin between primary GTV and its CTV was 0.5 cm. Circumferential margin around nodal GTVs and their CTVs was 1.0 cm. Per treatment protocol, uninvolved nodal stations were not targeted for prophylactic coverage, regardless of stage. Planning Target Volume (PTV) provided circumferential margin of 2 mm around each CTV to compensate for the variability in treatment set up and internal organ motion. All patients were prescribed 42.5 Gy to D95% of the PTV in 5 fractions.

**Table 1:** Study Cohort Characteristics

Patient #	Gender	Age	Primary site	Stage	GTV (cm^3^)
1	M	63	Glottic larynx	T1aN0	0.13
2	M	59	Glottic larynx	T2N0	6.49
3	M	79	Glottic larynx	T2N0	7.90
4	M	39	Glottic larynx	T1aN0	0.88
5	M	70	Glottic larynx	T2N0	3.08
6	M	75	Supraglottic larynx	T3N0	11.40
7	F	87	Glottic larynx	T4N0	12.10
8	M	78	Glottic larynx	T4N0	4.80
9	M	57	Glottic larynx	T3N0	8.20
10	M	68	Glottic larynx	T3N0	6.08

Avoidance structures included brainstem, spinal cord, parotid gland, carotid artery, and arytenoid cartilage. The spinal cord and the brainstem were expanded 5 mm to create a planning organ at risk volume (PRV). The maximum dose to spinal cord/brainstem, contralateral arytenoid, and contralateral carotid could not exceed 10 Gy, 21.4 Gy, 26.9 Gy, respectively. Maximum doses were calculated from a 0.035 cm^3^ subvolume. Mean dose to parotids could not exceed 26 Gy.

**Figure 1:**
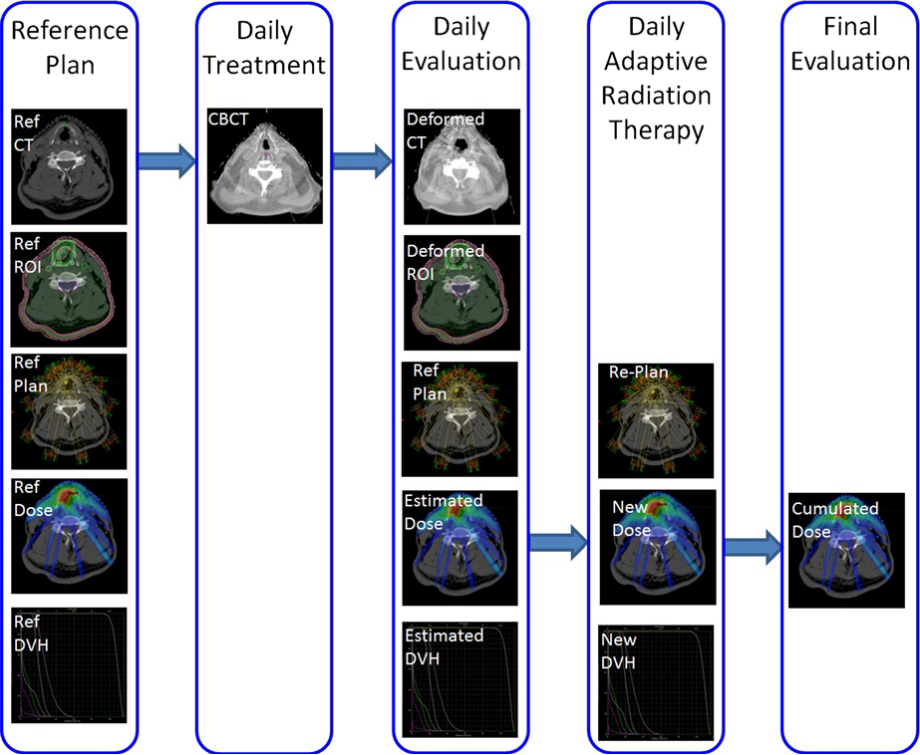
Adaptive replanning and SBRT QA flowchart.

**Figure 2:**
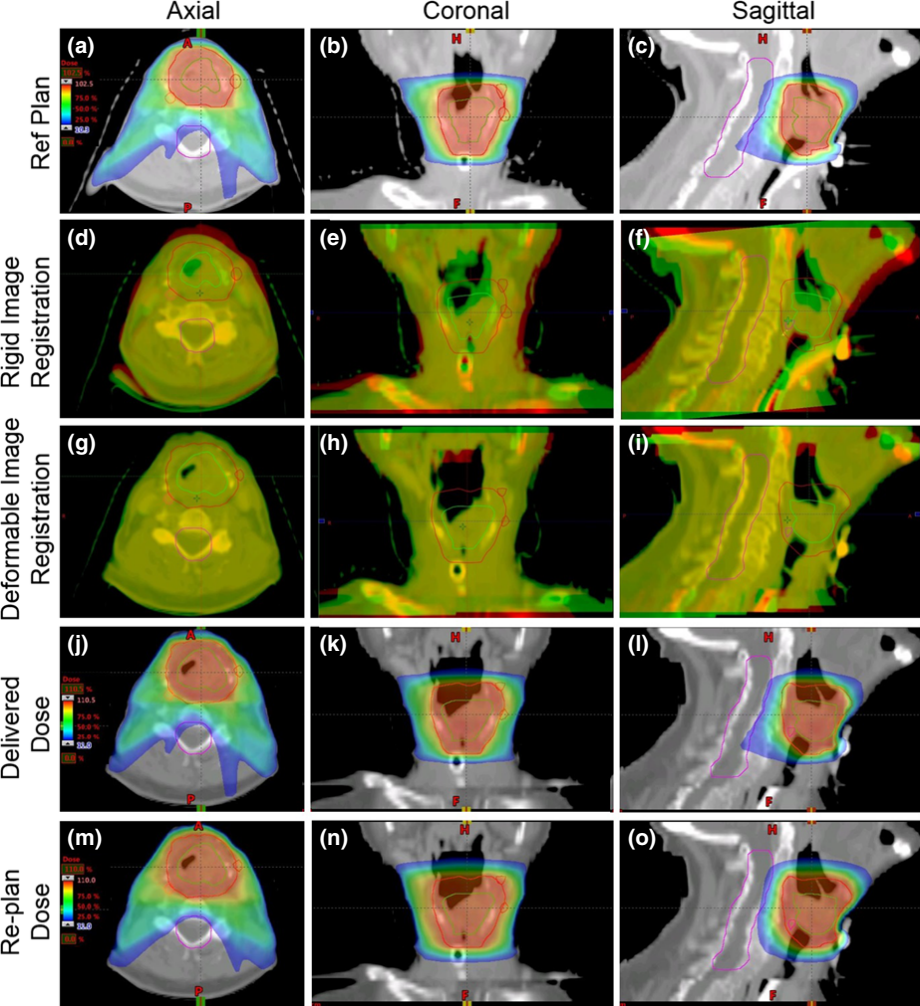
A representative example of our adaptive treatment planning process. (a)–(c) axial, coronal, and sagittal views of reference dose distribution on simulation CT images; (d)–(f) rigid image registration results; (g)–(i) deformable image registration results; (j)–(l) dose distribution of reference plan directly delivered to the deformed sim CT; (m)–(o) dose distribution on deformed sim CT after re‐planning.

We have established a SBRT quality assurance (QA) procedure to evaluate SBRT treatment delivery. All treatment planning and treatment delivery evaluation was performed with a commercial planning system (Eclipse v.11; Varian Medical Systems, Palo Alto, CA, USA) combined with in‐house software. Figure 1 illustrates a flowchart for our SBRT QA and adaptive replanning platform. All baseline SBRT treatment plans were generated in Eclipse per protocol directives. Figure 2 demonstrates images collected at specific phases along our adaptive replanning process from a representative case. Thermoplastic masks were used to immobilize patients from CT simulation to every fraction of treatment. The number of days between CT simulation to the first fraction of treatment varies from 3 to 33 days (average = 15.6 days). The number of days between consecutive fractions of treatment varies from 2 to 5 days with an average of 2.8 days. For one patient (#3) with travel issues there was an 11 day interval between the first and second fraction of treatment. Daily cone‐beam CT (CBCT) for Patients 6–10 or conventional fan‐beam CT images for Patients 1–5 were acquired prior to every treatment fraction. Reference simulation CT images and target volumes were deformably registered to daily images via commercially available software (SmartAdapt; Varian Medical Systems, Palo Alto, CA, USA). SmartAdapt uses “accelerated demons” algorithm.^16^ Figs. 2(g)–2(i) demonstrate an example of our deformable image registration results. A board‐certified medical physicist and a physician reviewed the registration results. The deformed simulation CT and contours were exported and reloaded in the Eclipse system and the reference SBRT plan was directly copied to deformed images and segmented volumes in Eclipse. Isocenters were always defined at mass centers of PTVs, and were shifted according to patient setup errors or deformably corrected daily imaging. Delivered dose distributions were obtained by re‐calculating doses on deformed CT images in Eclipse with Anisotropic Analytical Algorithm (version 11.0.31). Dose distributions were visually reviewed by a physician and a medical physicist. DVH curves were automatically checked by in‐house software in Matlab 2013b (Mathwork, Natick, MA, USA). Any plan could be re‐optimized with identical planning dosimetric constraints in Eclipse.

We have developed an in‐house software package to propagate all delivered dose distribution back onto reference CT simulation images to calculate cumulative doses. The package was developed first in Matlab and then in Visual Studio C++ 2010 (Microsoft, Redmond, WA, USA). The software loaded (1) dose distribution files, (2) reference structure files, and (3) deformable image registration results in DICOM format. It deformed radiation dose on 3 mm calculation grids from the daily images to reference CT simulation images using the Eclipse registration results (grid size = 4 mm). Deformed doses on irregular grids were resampled to a fine regular orthogonal grid using the half pixel size of reference CT images (pixel size typically = 1.12 mm) to create a dose‐volume histogram (DVH). Deformed dose accuracy was validated via a commercially available system (Mirada DBx; Mirada Medical USA, Denver, CO, USA). Mirada DBx loads reference simulation CT images, deformed CT images, recalculated dose distributions on deformed CT images, and deformable image registration results from Eclipses. It then deforms recalculated dose distributions back onto the reference CT images using Eclipse SmartAdapt registration results. Three‐dimensional Gamma analysis,^17^ maximum dose, DVH curves comparisons were performed to compare results from our in‐house software with those from Mirada.

## 3 Results

Performance of our online adaptive SBRT QA platform was relevant to the timeframe of routine clinical care. On average, for every fraction of treatment the software required 3 minutes for deformable image registration, 1 minute to map contours, 1.5 minutes for dose re‐calculation, about 1.5 minutes for data loading and exporting, and about 3 minutes for re‐planning. In sum, online adaptive replanning was completed from initial data input to final data export within 10 minutes for each treatment.

**Figure 3:**
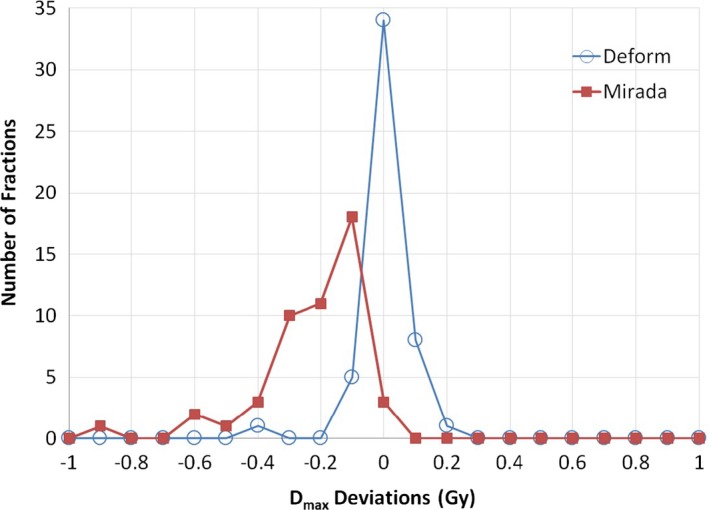
PTV Dmax error distributions resulting from our in‐house (Deform) and commercial (Mirada) dose deformation methods.

**Figure 4:**
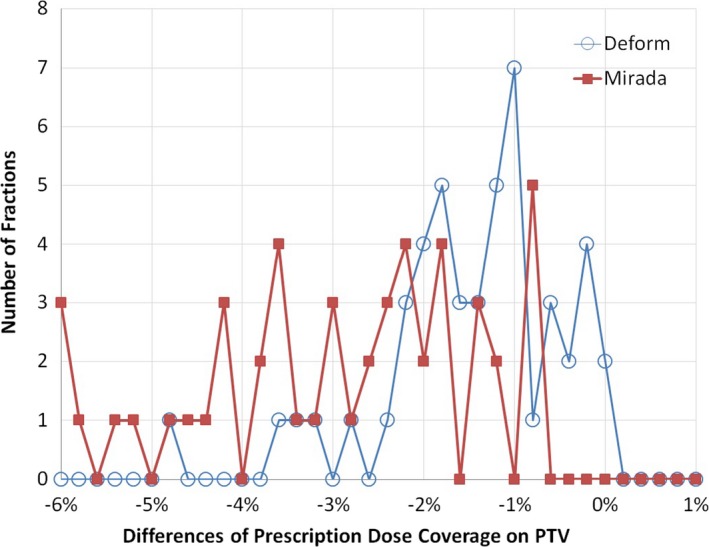
Respective calculation of PTV coverage deficiencies with our in‐house (Deform) and commercial (Mirada) dose deformation methods.

We compared our deformed dose distribution with results from commercial (Mirada) software via three‐dimensional Gamma analysis. For all fractions of delivery, the passing rate of 3% and 3 mm ranged from 97.23 to 99.99% (mean = 99.71%) while the passing rate of 3% and 1 mm ranged from 97.06 to 99.99% (mean = 99.59%). Large discrepancies occurred around the dose calculation boarders. The dose calculation box remained rectangular in both the reference plan and the adaptive replan in Miranda. To preserve efficiency, our in‐house software deforms representative tetrahedra containing the patient body, yielding some irregular external surface/boarders. The two algorithms also interpolate dose along calculation borders differently. In order to compare interpolation fidelity from dose calculation grids to CT voxels, we benchmarked our post‐deformation PTV Dmax calculations to Mirada. PTV Dmax was obtained on three sets of dose distributions, the delivered dose distribution (before the dose deformation), and two sets of deformed dose distributions by our dose deformation and Mirada method, separately. Detected differences were considered errors. PTV D_max_ error distributions for each method are shown in Fig. 3. Average Dmax dose errors were −0.02 Gy using our platform while average Dmax errors were −0.26 Gy for Mirada. DVH curves of deformed dose distributions were slightly different. PTV coverage was compared via average delivered PTV doses. Across all fractions of treatment, dose differences between our method and delivered dose ranged from −0.43 Gy to 0.50 Gy (mean = 0.06 Gy), while dose differences between Mirada results and delivered plans varied from −0.82 Gy to 0.07 Gy (mean = −0.26 Gy). PTV coverage deficiencies are summarized in Fig. 4. Our system calculated an average daily PTV coverage deficiency of 1.5%, while the commercial system calculated an average PTV coverage deficiency of 3.1%.

Delivered PTV dose coverage for all study cases were quantified and compared with prescribed doses using an in‐house Matlab program. The protocol required prescription dose cover 95% of PTV. Table 2 tabulates mean daily minimum, maximum, and average fractional PTV, CTV, and GTV prescription dose coverage for each patient. Prescribed treatment to GTV and CTVs was preserved in all patients. The minimum daily prescription dose coverage of CTV was 93.8% and total average CTV coverage was 98.3%. However, interfraction anatomic/set up changes led to loss of intended PTV dose coverage during 53% of individual treatment fractions. The average daily prescription dose coverage of PTV was 93.9% (range: 83.9–98.7%). One case (patient #7) had a single treatment where coverage of PTV dropped to 83.9%, although intended CTV coverage remained above 96.9%. This patient demonstrated significant interfraction motion. We could not reposition this patient for the first treatment fraction, and we thus re‐simulated the patient. A new reference plan based on the re‐simulation CT was generated and delivered for all planned treatments while large interfracton motions still occurred.

**Table 2:** PTVs, CTVs, and GTVs daily dosimetric outcomes

Patient #	PTV coverage			CTV coverage			GTV coverage		
	Min	Max	Avg	Min	Max	Avg	Min	Max	Avg
1	95.1%	97.9%	96.7%	97.8%	100.0%	99.1%	100.0%	100.0%	100.0%
2	96.2%	97.0%	96.6%	99.5%	99.9%	99.7%	100.0%	100.0%	100.0%
3	95.2%	96.9%	96.0%	99.6%	100.0%	99.7%	100.0%	100.0%	100.0%
4	93.2%	97.5%	96.0%	99.2%	100.0%	99.8%	100.0%	100.0%	100.0%
5	91.1%	95.6%	93.8%	99.4%	99.7%	99.5%	99.8%	100.0%	100.0%
6	88.6%	92.0%	90.6%	96.8%	97.8%	97.3%	100.0%	100.0%	100.0%
7	83.9%	92.1%	89.2%	96.9%	98.5%	97.6%	99.2%	100.0%	100.0%
8	91.0%	93.9%	92.6%	93.8%	94.5%	94.2%	99.3%	99.9%	99.7%
9	93.8%	97.8%	95.4%	97.0%	99.3%	98.1%	97.5%	99.0%	98.3%
10	90.9%	93.5%	92.4%	97.3%	98.2%	97.7%	99.6%	100.0%	99.8%

Mean cumulative dose coverage of CTV and GTV was 97.9% (range: 95.2–99.5%) and 99.8% (range: 98.3–100.0%), respectively (Table 3). Cumulative PTV dose coverage was less favorable across the study cohort. Mean coverage of PTVs with prescribed dose was 92.7% (range: 86.1–96.1%). D95% PTV coverage was 39 Gy or greater in all cases. Cumulative PTV V_Rx_ for Patient #7 was 86.1%.

**Table 3:** Cumulative PTV, CTV, and GTV prescription dose coverage

Patient #	PTV	CTV	GTV
1	94.9%	98.0%	100.0%
2	96.1%	99.5%	100.0%
3	95.2%	99.5%	100.0%
4	94.9%	99.0%	100.0%
5	93.0%	99.4%	100.0%
6	89.1%	95.6%	100.0%
7	86.1%	97.6%	99.9%
8	92.1%	95.2%	99.9%
9	95.1%	98.3%	98.3%
10	90.8%	97.4%	99.9%

**Figure 5:**
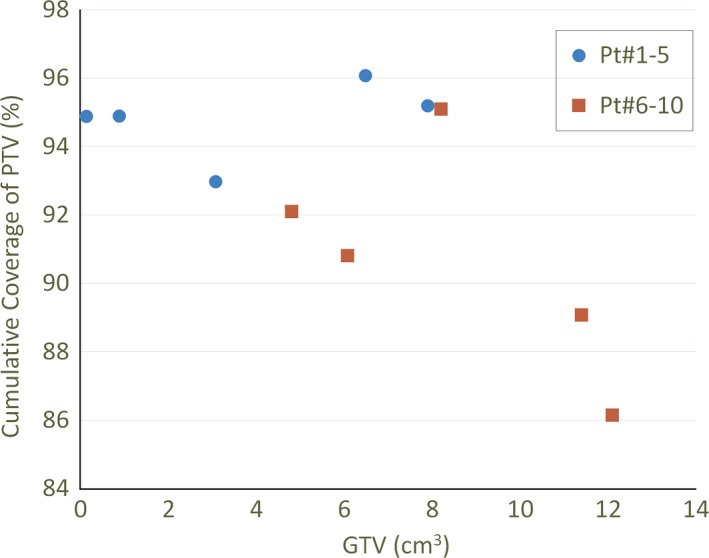
Cumulative PTV coverage as a function of GTV volume.

Our patients came from two studies with different advanced stages of cancer. Advanced stage disease (Pt# 6–10) with larger GTV and disease spread outside the laryngeal cartilage indeed makes the balance between PTV coverage and OAR sparing more challenging to achieve. Figure 5 illustrates the PTV prescription dose coverage as a function of GTV volume and it shows that coverage decreases with GTV volume.

Changes in bystander dose delivery to organs at risk (OARs) are detailed in Table 4. Maximum point dose and Dmean to arytenoids, spinal cord, and parotids were well respected in all cases. Cumulative Dmax to a single carotid artery increased by 13.9 Gy (patient #3) and 6.5 Gy (patient #2) from intended doses in two patients. Dmax > 45 Gy were observed for several OARs which overlapped with PTV.

**Table 4:** Differences (Diff) between cumulative delivered (Cumul) and reference prescription doses (Ref) to organs at risk

Pt #	Rt carotid D_max_ (Gy)			Lt carotid D_max_ (Gy)			Contral arytenoid D_max_ (Gy)			Spinal cord D_max_ (Gy)			Rt parotid D_mean_ (Gy)			Lt parotid D_mean_ (Gy)		
	Ref	Cumul	Diff	Ref	Cumul	Diff	Ref	Cumul	Diff	Ref	Cumul	Diff	Ref	Cumul	Diff	Ref	Cumul	Diff
1	12.6	12.1	−0.5	9.9	9.0	−0.9	12.5	16.1	3.6	8.8	9.0	0.2	0.3	0.4	0.1	0.3	0.4	0.1
2	20.9	27.4	6.5	22.2	23.7	1.5	16.7	21.7	5.0	8.4	10.3	1.9	0.2	0.2	0.0	0.2	0.3	0.1
3	27.9	41.8	13.9	23.4	30.6	7.2	—	—	—	6.7	7.9	1.2	—	—	—	—	—	—
4	13.4	15.6	2.2	7.3	7.9	0.6	14.0	14.4	0.4	8.1	8.1	0.1	—	—	—	—	—	—
5	10.9	10.7	−0.3	8.0	8.5	0.5	18.3	20.6	2.2	8.6	6.9	−1.8	—	—	—	—	—	—
6	16.8	17.7	0.9	15.1	14.2	−0.9	22.3	19.0	−3.4	5.7	4.8	−0.9	—	—	—	—	—	—
7	44.7	44.4	−0.3	44.8	44.6	−0.2	44.5	45.0	0.4	9.9	11.3	1.3	—	—	—	—	—	—
8	13.2	16.1	2.9	12.1	12.0	−0.1	45.1	45.6	0.5	8.1	8.9	0.8	0.4	0.4	0.0	0.3	0.3	0.0
9	13.1	13.1	0.0	43.3	42.1	−1.1	45.1	44.8	−0.4	7.8	7.8	−0.1	0.5	0.5	0.0	0.8	1.1	0.3
10	23.7	23.6	−0.1	20.4	19.7	−0.7	45.3	44.6	−0.6	2.3	2.0	−0.3	0.4	0.3	0.0	0.3	0.3	0.0

## 4 Discussion

Hypofractionation promises improved disease control (particularly for advanced disease stage presentations), cost savings, and patient convenience for definitive treatment of laryngeal cancer. Our findings support continued prospective testing of adaptive replanning to optimize the quality and safety of this approach. Dosimetric assessment could be performed within 10 minutes and leveraged straightforward in‐house software to supplement a commercially available planning platform. Adaptive replanning can potentially prevent PTV coverage failure along steep SBRT dose gradients in up to half of cases.

SBRT requires tight geometric tolerances to maintain safety of large fraction sizes and sharp dose gradients. Key priorities include prevention of overdosing along unanticipated overlaps of adjoining PTVs that drift toward one another, and prevention of underdosing due to tumor migration out of a high dose target volume. Our patients had in‐room CBCT imaging performed prior to each fraction for image‐guided setup. Nonetheless, our post hoc dosimetric analysis revealed that prescribed dose coverage to PTV was lost during more than half of SBRT treatments. Correction of acute dose deficiencies during individual fractions (as in the case of patient #7) is unlikely with routine IGRT‐based positional correction unaccompanied by replanning. Given that GTV and CTV dose coverage was not lost in any case, the downstream clinical consequences of PTV coverage deficiencies remain undefined. Nonetheless, optimal therapeutic ratio requires preservation of intended tumor coverage and sparing of neighboring OARs. We, in fact, observed unanticipated Dmax dose increases to carotid arteries and contralateral arytenoids in uncorrected cases where these OARs bordered PTVs (Table 4). This potentially supports judicious use of PRVs as a planning technique to protect specific critical normal structures in individual cases, although adaptive replanning would more globally address this and other longitudinal dosimetric safety issues.

Taken together, our in‐house ART software potentially provides valid QA and treatment quality support at clinically relevant speed. However, several factors yield uncertain impact on the platform's accuracy. First, the ideal algorithm to interpolate deformable image registration and dose calculation results from a coarse grid (~4 mm) to finer resolutions (~0.56 mm) remains unclear. Second, DVH calculations are dependent upon specific edge detection techniques used to delineate volumes of interest;^18^ an optimal solution remains unidentified. Third, inverse application of Eclipse's deformable image registration results may lead to further dose uncertainties.^19^ These remain active areas of investigation for platform enhancement.

## 5 Conclusions

Our online adaptive SBRT replanning platform appears accurate and relevant to routine clinical care. Although GTV and CTV dose coverage can be preserved with CT‐based IGRT guidance throughout a course of hypofractionated treatment, PTV coverage can vary significantly from intended plans. Use of online adaptive treatment re‐planning is potentially necessary and clinically feasible for preserving the quality of head and neck SBRT. Formal validation of the feasibility and downstream clinical impact of adaptive replanning will require continued prospective analysis. Patient accrual, dosimetric analysis, and software refinement remain ongoing.

## Acknowledgment

This project was supported by CPRIT Individual Investigator Research Award RP150386.

## Conflict of interest

The authors report no relevant conflicts of interest.
